# Endothelial cell clonality, heterogeneity and dysfunction in pulmonary arterial hypertension

**DOI:** 10.3389/fmed.2023.1304766

**Published:** 2023-12-06

**Authors:** Geoffrey Newcomb, Laszlo Farkas

**Affiliations:** ^1^Department of Internal Medicine, Division of Pulmonary, Critical Care, and Sleep Medicine, The Ohio State University Wexner Medical Center, Columbus, OH, United States; ^2^Davis Heart and Lung Research Institute, The Ohio State University, Columbus, OH, United States

**Keywords:** EPCs, endothelial, pulmonary hypertension, endothelial dysfunction, cell heterogeneity

## Abstract

Our understanding of the pathophysiology of pulmonary arterial hypertension (PAH) has evolved over recent years, with the recognition that endothelial cell (EC) dysfunction and inflammation play an integral role in the development of this disease. ECs within the pulmonary vasculature play a unique role in maintaining vascular integrity and barrier function, regulating gas exchange, and contributing to vascular tone. Using single-cell transcriptomics, research has shown that there are multiple, unique EC subpopulations with different phenotypes. In response to injury or certain stressors such as hypoxia, there can be a dysregulated response with aberrant endothelial injury repair involving other pulmonary vascular cells and even immune cells. This aberrant signaling cascade is potentially a primary driver of pulmonary arterial remodeling in PAH. Recent studies have examined the role of EC clonal expansion, immune dysregulation, and genetic mutations in the pathogenesis of PAH. This review summarizes the existing literature on EC subpopulations and the intricate mechanisms through which ECs develop aberrant physiologic phenotypes and contribute to PAH. Our goal is to provide a framework for understanding the unique pulmonary EC biology and pathophysiology that is involved in the development of PAH.

## 1 Introduction

In most organs, endothelial cells (ECs) play a central role as a main component of the capillaries to provide oxygen and nutrition to the parenchymal cells (1). In the lung however the function of ECs is more complex, as the vascular system has dichotomous functions compared to any other vascular system in the body (2). The lung has two separate vascular systems: the bronchial circulation that supplies the airways and pulmonary circulation, which accounts for the unique features of the lung vasculature. In this circulatory system, the right heart ejects deoxygenated blood towards the lung via the arterial branch of the pulmonary circulation. Upon passage of the pulmonary capillary system, CO_2_ and O_2_ are exchanged with the ventilation air inside of the alveolus. At every stage of the process, ECs play a crucial role in regulation of the function of the pulmonary vascular system, e.g., by regulation of vasotonus and regulation of EC barrier.

## 2 Endothelial cell heterogeneity

While a distinction between main anatomic compartments in the lung has also been made by separating pulmonary arteries, microvascular (capillary) and pulmonary veins in the pulmonary circulation (2), little thought has been given to the potential heterogeneity of ECs within these anatomic localizations until recent years. At the same time, the assumption that all ECs derived from a single anatomic compartment, such as pulmonary artery, have identical functional properties has long persisted, even though more recent work has already shown heterogenous responses within a seemingly homogenous population. As an example, work by Sakao et al. (3, 4) identified that exposure of lung microvascular cells to SU5416 results in initial apoptosis followed by unchecked proliferation. When dismissing this as a compensatory proliferation process, it does not explain why only a fraction of ECs undergo apoptosis, and the surviving cells express progenitor markers and undergo extensive proliferation. Alternatively, these cultured ECs may contain multiple cell clusters or even a hierarchy of progenitor cells as suggested by Yoder and others (5, 6), with distinctive population of pro-proliferative stem or progenitor cells adjacent to clusters of apoptosis-prone ECs.

Recent technological advances have made their way into pulmonary vascular research and substantially enhanced our understanding of cellular heterogeneity. Separate populations were described, e.g., in the pulmonary microvasculature based on single-cell transcriptomics. Gillich et al. (7) showed a specialized EC population that contains pores and is spread out for gas exchange. They labeled this EC population “aerocytes,” now commonly used (8–10). Similar to lung alveolar epithelial cells, there is not only a specialized EC population responsible for gas exchange, but the lung microvasculature contains at least another EC population with progenitor/stem cell-like properties which could be responsible for vascular regeneration, generally capillary ECs (gcap) (7–10). Progenitor ECs help explain the substantial regenerative potential of the pulmonary vasculature, and in this regard, neonatal lung angiogenesis driven by progenitor ECs requires FoxF1-mediated activation of bone morphogenetic protein-9 (BMP9) and activin A receptor-like 1 (ACVRL1) signaling (11). Interestingly, aerocytes and gcap ECs express complementary patterns of growth factors and their receptors (7). As an example, aerocytes express high mRNA levels of stem cell factor, whereas gcap ECs express the receptor, c-kit. Our own work has shown that blocking stem cell factor in c-kit (CD117) expressing lung ECs, assumed to overlap with the gcap population, reduced angiogenesis and cell viability (12). In contrast, the expression of the angiogenic factor apelin-1 and its receptor is opposite between gcap ECs and aerocytes compared to stem cell factor (7). Hence, there is not only specialization among the lung endothelium but also a potentially active crosstalk through growth factors that can influence endothelial maintenance and regeneration. How the endothelial populations in different sections of the pulmonary vascular tree contribute to the remodeling process in PAH will need further consideration.

## 3 Endothelial cell turnover and regeneration

Despite the central role of ECs in vascular homeostasis, surprisingly little is known about the rate of endothelial turnover and the detailed mechanisms governing its regeneration following injury (1). The existing body of literature suggests that ECs favor the stability of the endothelial monolayer with a mostly quiescent state under physiologic conditions and a well-regulated repair mechanism when the need arises (1, 13). There is little data on the turnover rate of lung endothelium, but the available data suggest that the turnover of ECs is rather low compared to epithelial cells and immune cells and is consistent with ECs in other organs (14, 15). Yet the proximity of ECs to the outside environment places lung ECs in a position that is more prone to injury due to the exposure to environmental factors during breathing. This could explain the presence of immature or progenitor-like ECs in the lung microvascular endothelium which may be as high as 50% of the microvascular endothelium and potentially allow for rapid expansion and regeneration (16). The unusual functional and anatomic context of the lung endothelium could also explain the potential endothelial origin of a condition with abnormal endothelial repair – PAH (17). PAH represents an array of consequences of aberrant endothelial injury repair that not only involves other pulmonary vascular cells but also immune cells and bone marrow-derived cells. The mechanism of how endothelial injury leads to this complex condition is still poorly understood; however, research into this is ongoing. FoxM1 is a transcriptional factor that promotes lung endothelial regeneration following lung tissue and vascular injury (18, 19). FoxM1 is also activated in the context of PH development to stimulate endothelial and pulmonary artery smooth muscle cell (PASMC) proliferation. In addition, research suggests a role for FoxM1 in regulation of the paracrine function of ECs and the development PAH. In this regard, PAH EC-conditioned growth medium promotes proliferation of PASMCs likely driven by FoxM1 and the production of paracrine factors endothelin-1, C-X-C motif chemokine ligand 12 (CXCL12), fibroblast growth factor-2 (FGF2), platelet derived growth factor B (PDGFB), and macrophage migration inhibitory factor (MIF) (20, 21).

## 4 Role of clonal expansion in physiologic and pathologic endothelial cell function

Clonal expansion is mostly associated with the rapid expansion of cells in specific populations, such as in various types of cancers (22–24). Sometimes, the physiologic role of clonal expansion is disregarded although it is evident in various tissues. The best-known example of clonal expansion occurs in the adaptive immune system, where selection and expansion of specific clones of T cells and B cells is a fundamental mechanism for fostering an immunological memory (25, 26). ECs are commonly seen as more static with a low cell turnover, which is likely a physiologic necessity to maintain a consistent smooth vascular lumen. However, at the same time ECs require the ability to quickly regenerate if needed to avoid complications such as thrombosis and vascular inflammation (17, 27, 28). Clonal expansion is a logical and quick way to increase the cell numbers upon injury without the need to keep many actively proliferating progenitors on site, which may complicate maintaining a well-regulated endothelial monolayer. Hence, ECs throughout the body use clonal expansion as mechanism for regeneration following vascular injury (24). Early *in vitro* experiments have shown that pulmonary EC injury combined with a second factor, such as high shear stress leads to exuberant proliferation of the surviving endothelial cells which exhibit a progenitor cell phenotype (3, 4). In PAH, it was postulated that monoclonal expansion of primitive, stem-like ECs could be the origin of ECs in plexiform lesion in idiopathic PAH (29). We have recently demonstrated that clonal expansion of primitive CD117^+^ rat lung ECs yields stem-like EC clones with exaggerated proliferative capacity and increased susceptibility to mesenchymal transition (12). Upon transplantation to rats and exposure to chronic hypoxia, an occlusive pulmonary arteriopathy formed in these rats. One potential driver of this phenotype of abnormal proliferation was the downregulation of the master regulator of the cell cycle, p53, following multiple rounds of clonal expansion (30). Hence, pathogenic clonal expansion with loss of p53 expression could be a potential underlying mechanism for the development of occlusive lesions in PAH.

## 5 Immune dysregulation and endothelial function

Inflammation and immune dysregulation are known drivers of the pathogenesis of PAH. Certain autoimmune diseases share a predilection for the development of PAH, such as systemic sclerosis (31), mixed connective tissue disease, and systemic lupus erythematosus (Tselios). Circulating serum levels of certain cytokines (e.g., interleukin-1β [IL-1β] and IL-6) and chemokines (CC chemokine ligand 2, Regulated upon Activation, Normal T Cell Expressed and Presumably Secreted [RANTES]) are elevated in idiopathic PAH (32–34). There are also increases of various cytokines and growth factors in certain PAH subtypes, such as IL-1α and IL-6 in connective tissue disease associated PAH (35), platelet-derived growth factor in human immune deficiency virus-associated PAH (36), and tumor necrosis factor-α (TNF-α) and IL-6 in congenital heart disease-associated pulmonary hypertension (PH) (37). PAH patients with elevated serum levels of IL-6 had worse 5-year survival compared with those with lower levels of IL-6 (38). Additionally, PAH patients with reduced 6-min walk distances had elevations in multiple cytokines, such as IL-1β and IL-6 (39); however, other studies have not found a clear correlation between increase in these cytokines and other hemodynamic parameters (33, 38). There is also significant perivascular inflammatory cell infiltrate and EC hyperproliferation in the classic plexiform lesions seen in PAH patients (40). Although plexiform lesions are seen in most patients with advanced PAH, presence of these lesions do not directly correlate with other hemodynamic parameters of disease severity (41). Since these plexiform lesions are generally seen in the later, severe stages of disease, unchecked inflammation is likely a persistent, driving feature of PAH pathogenesis and endothelial dysfunction.

On a cellular level, there is further evidence of immune dysregulation and alterations in the innate immune system. ECs are the first line of defense for the vascular system against invading pathogens. ECs will recognize pathogen-associated molecular patterns (PAMPs) as well as damage-associated molecular patterns (DAMPs) which initiates downstream upregulation of type I interferons and activation of inflammatory transcription factors. In PAH, there appears to be aberrant immune signaling within ECs leading to apoptosis selection of apoptosis-resistant ECs, and proliferation of nearby vascular smooth muscle (4, 42, 43). The Toll-like receptor (TLR) family is an ancient, highly conserved group of receptors involved in the innate immune response and are responsible for recognition of PAMPs and DAMPs. This then leads to upregulation of various inflammatory cytokines and interferons such as IL-6, CXC chemokine ligand 8 (CXCL8), and CC chemokine ligand 2 (CCL2) (44, 45) We have previously shown that expression of TLR3, an endosomal RNA receptor, is reduced in ECs and lung tissue of patients with PAH and that TLR3^−/−^ mice exhibit more severe pulmonary hypertension (42). Activation of this dsRNA receptor system with the administration of polyinosinic: polycytidylic acid (PolyI:C), a synthetic dsRNA, recovered TLR3 levels within ECs in an IL-10 dependent manner. High dose PolyI:C was also shown to prevent and reverse established PAH in a rat model of progressive pulmonary hypertension (chronic hypoxia and SU5416). TLR3 transcription within ECs is regulated by p53 and downstream TLR3 signaling via IRF3 regulates transcription of bone morphogenic protein receptor 2 (BMPR2) via direct binding of IRF3 on the BMPR2 promoter, hence linking cell cycle regulation via p53, regulation of innate immunity and BMPR2 signaling as a novel axis driving pulmonary artery remodeling and PAH (30).

In addition, researchers have also examined the role of anti-inflammatory cytokines in the pathogenesis of PAH. Patients with PAH have elevated levels of circulating IL-10, which were higher in patients receiving prostacyclin therapy and correlated with reduced survival (38). Interestingly, systemic overexpression of IL-10 in a monocrotaline rat model protects from PAH development (46). Thus, it stands to reason that the upregulation of IL-10 represents a compensatory response and it remains to be studied if an impaired cellular response to these elevated IL-10 levels is a contributing factor in PAH.

There is additional evidence that regulation of inflammation protects against the development of PAH. Regulatory T-cells have been shown to play a key protective role in the development of PAH. Administration of either monocrotaline or SU5416 to induce widespread EC apoptosis and injury leads to the development of pulmonary hypertension in athymic mice without exposure to hypoxia (47, 48). Likewise, PAH development with monocrotaline is attenuated with immune reconstitution in these athymic, T-cell deficient rats (49). The Forkhead box protein O (FoxO) transcription factors maintain vascular integrity and prevention of SMC hyperproliferation (50–52). The expression of FoxO1 is reduced with exposure to inflammatory cytokines, such as IL-6 (53). FoxO1 expression is downregulated in the pulmonary vasculature and PASMCs of patients with PAH, and reconstitution of FoxO1 activity reverses vascular remodeling and right ventricular hypertrophy *in vitro* (54).

## 6 Mutations regulating endothelial function

The best-studied genetic mutation associated with the development of PAH is an alteration in *BMPR2*, the gene encoding bone morphogenetic protein receptor type 2, which is a member of the transforming growth factor-β (TGF-β) superfamily (55, 56). The BMPR2 protein is highly expressed in the pulmonary vascular endothelium and loss of BMPR2 has been shown to promote EC apoptosis and endothelial-to-mesenchymal transition (57–59). The impact of EC mutations in *BMPR2* is further supported by connections between mutations in other genes involved in the BMPR2 signaling pathway, EC dysfunction, and the development of PAH. BMPR2 forms a complex with activin A receptor like type 1 (ALK1), a transmembrane serine/threonine receptor kinase, on the surface of endothelial cells. When this complex binds to BMP ligands – BMP9 and BMP10 – along with the coreceptor, endoglin (ENG), it leads to downstream activation of the SMAD family member (SMAD) signaling pathway (60, 61). ALK1 and ENG also have significant levels of expression within the pulmonary endothelium and mutations in each of these proteins have been linked to the development of PAH as well as hereditary hemorrhagic telangiectasia (HHT) (56, 62, 63). Previous studies have shown that a mutation in *BMPR2* is the cause of PAH in 70–80% of those with familial PAH and in 10–20% of IPAH patients. Patients with PAH and a *BMPR2* mutation generally present at a young age with more severe disease and increased risk of death (64). Although this association is significant, the actual penetrance of this mutation remains quite low, around 20%, suggesting additional underlying pathophysiology is contributing to PAH development (65). A meaningful contribution of environmental factors is likely to PAH development given the small subset of genetic mutations that have been linked to congenital and early onset of PAH. Mutations in *TBX4*, a gene that encodes a transcription factor necessary for lung development, and *ATP13A3*, a gene which encodes a polyamine transporter, are linked to childhood-onset of PAH (66–68).

Hypoxia signaling is another important contributor to endothelial dysfunction in PAH. Chuvash polycythemia develops with a specific missense mutation in von Hippel–Lindau tumor suppressor (*VHL*), which reduces its binding to hydroxylated hypoxia inducible factor-α (HIF-α) subunits and increases levels of HIF-1α and HIF-2α (69, 70). HIFs are heterodimeric transcription factors which bind to hypoxia response elements (HREs) within target genes when cells are exposed to hypoxic conditions. The two primary HIFs are HIF-1α and HIF-2α and play unique roles in the development of PAH. They are regulated by prolyl hydroxylases (PHD) – particularly PHD2 – and by factor inhibiting HIF (FIH). HIF-1α is ubiquitously expressed while HIF-2α is highly expressed in ECs. With various loss of function mutations in *VHL*, patients were found to have significantly higher resting pulmonary artery pressures, severe pulmonary hypertension, and right ventricular dysfunction (71, 72). A recently published genome-wide association study highlighted an overrepresentation of mutations in certain genes of PAH patients, one of which is SRY-box 17 (*SOX17*) (73). SOX17 is an endothelial-specific transcription factor involved in angiogenesis and acts as a positive feedback regulator of vascular endothelial growth factor (VEGF) signaling (74). Downregulation of SOX17 in pulmonary artery ECs (PAECs) increases susceptibility to PAH, particularly with exposure to hypoxia (75, 76). Notably, mice with EC-specific SOX17 deletion developed spontaneous, mild PH and exacerbated hypoxia-induced PH (77). Upregulation in SOX17 can also attenuate PAH via inhibition of HIF2α (78).

## 7 Conclusion

In conclusion, the specialized and diverse function of the pulmonary endothelium is represented by newly discovered subpopulations. Among these populations, there are differences in the proliferative capacity which can lead to aberrant EC proliferation and development of PAH following certain physiologic stressors or due to mutations involved in these pathways. A better understanding of these mechanisms will allow researchers to develop novel, targeted therapies for this devastating, lethal disease.

## Author contributions

GN: Conceptualization, Writing – original draft, Writing – review & editing. LF: Conceptualization, Writing – original draft, Writing – review & editing.
